# Tract-specific analysis of diffusion MRI at 3T detects cervical spinal cord aberrations in multiple sclerosis

**DOI:** 10.1162/IMAG.a.72

**Published:** 2025-07-07

**Authors:** Atlee A. Witt, Sawyer Fleishman, Delaney Houston, Logan E. Prock, Grace Sweeney, Trey McGonigle, Simon Vandekar, Maxime Chamberland, Seth Stubblefield, Colin D. McKnight, Kristin P. O’Grady, Kurt Schilling, Seth A. Smith

**Affiliations:** Vanderbilt University Institute of Imaging Science, Vanderbilt University Medical Center, Nashville, TN, United States; School of Medicine, Vanderbilt University, Nashville, TN, United States; Department of Biostatistics, Vanderbilt University Medical Center, Nashville, TN, United States; Department of Mathematics and Computer Science, Eindhoven University of Technology, Eindhoven, The Netherlands; Department of Radiology and Radiological Sciences, Vanderbilt University Medical Center, Nashville, TN, United States; Department of Biomedical Engineering, Vanderbilt University, Nashville, TN, United States

**Keywords:** spinal cord, magnetic resonance imaging, multiple sclerosis, diffusion tensor imaging, tractography

## Abstract

In people with multiple sclerosis (MS), diffusion tensor imaging (DTI) applied in the spinal cord (SC) can reveal microstructural damage prior to significant symptom burden, yet lacks specificity. Diffusion tensor tractography goes beyond conventional DTI-derived microstructural indices and, though widely applied in the brain, has yet to be fully exploited in the SC of people with relapsing-remitting MS (pwRRMS). We sought to determine the course of streamlines within cervical SC white matter (WM) fiber tracts in 46 healthy controls (HCs) and 56 pwRRMS scanned at 3T and investigate whether tractography provides added value beyond conventional analyses. We extended conventional lesion load analysis by investigating lesion load specific to white matter columns and streamlines within white matter columns, and quantified diffusion features along streamlines within a column. We identified reduced fractional anisotropy (FA) within WM columns for females and older participants, though these findings were more limited in WM streamlines. We also determined no significant associations between clinical variables and either lesion load or microstructural indices as derived from streamlines. While tractography in the MS SC may have value for visualization purposes, we did not determine added benefit to applying tractography compared to conventional volume or voxel-based analyses.

## 1 Introduction

Multiple sclerosis (MS) is one of the most common neurologic diseases affecting young adults, characterized by central nervous system (CNS) demyelination and axonal loss. Spinal cord (SC) MRI is recommended for suspected MS cases and offers valuable diagnostic insights, especially as SC lesions can help confirm CNS dissemination and have been associated with a greater risk of relapse and disease progression (Moccia et al., 2019; Trip, 2005). SC lesions are prevalent in MS, with some sources suggesting SC abnormalities in upward of 90% of people with MS (pwMS), and robust identification and quantification of lesions are, thus, critical to encompass the full spectrum of the disease (Bussas et al., 2022; Ruggieri et al., 2023). Despite supporting a diagnosis of MS, T2-weighted MRI lesion load does not always correlate with the diversity and severity of clinical presentation in MS often referred to as the clinico-radiological paradox (Chard & Trip, 2017). Conventional MRI techniques such as T1- and T2-weighted imaging are not sensitive enough to detect microstructural changes associated with both diseased and normal-appearing tissue in pwMS (Filippi et al., 2013). Further characterization of tissue damage requires advanced quantitative MRI, such as diffusion tensor imaging (DTI), that can capture both macrostructural lesion changes as well as microstructural alterations in tissue integrity in normal-appearing tissue beyond overt lesions.

DTI is sensitive to both the magnitude and direction of water molecule diffusion in tissue, where DTI-derived indices can describe tissue microstructure and serve as surrogate measures of physiological conditions (Alexander et al., 2007; Schmierer et al., 2007). Diffusion-derived microstructural indices include fractional anisotropy (FA), a measure of anisotropic water diffusion, and mean, axial, and radial diffusivities (MD, AD, RD) that represent either average magnitude (MD) or direction (AD/RD) of diffusion (Kolasa et al., 2019). In the brain of pwMS, increased RD has been posited to be associated with loss of myelin, while decreased FA is believed to be related to inflammation and reduced anisotropy typical of lesioned tissue (Fink et al., 2010; Schmierer et al., 2007). More specifically, people with relapsing-remitting MS (pwRRMS) demonstrate significantly increased RD and decreased FA in both normal-appearing white matter (NAWM) and lesions of the brain compared to healthy controls (HCs) with support from myelin sensitive measures in post-mortem histology (Ciccarelli et al., 2008; Kolasinski et al., 2012; Lin et al., 2007). These notable differences in brain DTI microstructural indices appear to be consistent in the SC of pwMS, with similar reductions in FA in lesions and NAWM compared to HCs (Jeantroux et al., 2012; Ohgiya et al., 2007). Combes et al. showed that FA was decreased and RD was increased in NAWM in the SC of pwRRMS when compared to HCs, indicating both a decrease in fiber coherence and the presence of demyelination (Combes et al., 2022). Despite the evident utility of applying DTI and deriving microstructural indices that are *sensitive* to many aspects of tissue microstructural damage, these indices have limited *specificity* to the underlying pathology. For instance, FA may be altered by edema, a phenomenon common to many disease states, and thus cannot be used in isolation to identify the specific processes underlying disease changes (Hori et al., 2022).

In addition to indices derived from a diffusion experiment, DTI-derived tractography is a unique approach to provide increased anatomical and even location-specific microstructural information. Tractography produces 3D representations of streamlines—or visual representations of likely axonal trajectories—that comprise a WM fiber tract (Saliba et al., 2020). While qualitatively meaningful, tractography’s true power lies within its ability to provide information about the extent and magnitude of tract damage when combined with microstructural information and specific lesion location. When applied in MS, DTI tractography in concert with diffusion-derived microstructural indices allows for quantification of microstructural alterations, such as decreased FA in ipsi-lesional WM fibers—in pwMS with lesions found unilaterally at the inferior cerebellar peduncle—and contra-lesional NAWM relative to HCs (Droby et al., 2015). Tractography can be further parsed into more spatially specific descriptors, including “bundles” to represent the full volume of WM streamlines in an anatomical tract, “streamline bundle load” as the volume of streamlines impacted by lesions divided by the volume of the bundle itself, “tractometry” as the sampling of diffusion-derived indices along each bundle (Bells et al., n.d.), and “lesionometry” as the sampling of diffusion-derived indices along bundle streamlines traversing a lesion (Chamberland et al., 2021). In combining indices with cognitive scores to better understand learning and recall differences in pwRRMS, Winter et al. determined tract-specific measures in the brain outperform traditional lesion load analysis and offer a promising mechanism to better understand neuropathology in MS (Winter et al., 2021).

The anatomy of the SC lends itself well to tractography with clear WM/gray matter (GM) delineation, including dorsal and ventral GM horns and anterior (ventral), lateral, and posterior (dorsal) columns in the WM. Though diffusion tensor tractography has been employed in amyotrophic lateral sclerosis, SC injury, and intradural SC tumors (Choe et al., 2017; Pisharady et al., 2020; Vargas et al., 2008), it has not yet been fully utilized for the purpose of better understanding MS pathology in the SC at 3T MRI field strength. Studies using either a probabilistic tractography algorithm in pwMS (Ciccarelli et al., 2007) or a region-based voxel-wide approach in people with demyelinating disease (Naismith et al., 2013) determined tract-specific alterations in SC diffusion indices and associations with disability, including between Expanded Disability Status Scale (EDSS) and RD of the corticospinal tracts. As DTI tractography has demonstrated merit in the SC, we sought to expand this analysis via MRtrix and compare whole-column with tract-specific indices as quantitative measures of SC tissue integrity.

To summarize, we aimed to apply diffusion tensor tractography in the SC of pwRRMS to highlight WM fiber tracts and group streamlines into six defined bundles (left ventral, left lateral, left dorsal, right ventral, right lateral, right dorsal) before extracting DTI indices along each bundle. We also sought to explore how those streamlines were influenced by lesions to draw associations with measures of disability, such as EDSS, Timed 25-Foot Walk (T25w), and Timed Up and Go (TUG) exam.

## 2 Methods

### 2.1 Data acquisition

Collection of anatomical and DTI data was approved by the Vanderbilt Institutional Review Board Health Sciences Committee and performed in accordance with relevant ethical guidelines and regulations. Inclusion criteria for pwMS included greater than 18 years old, EDSS score of less than or equal to 4, relapsing-remitting disease, no steroid treatment within 6 months prior to the scan, and no contraindications to 3T MRI. Inclusion criteria for HCs included greater than 18 years old, no SC injury, no history of neurodegenerative or demyelinating conditions, not currently pregnant if female, and no prior medical conditions precluding MRI.

HC and MS participants were asked to perform sensorimotor testing, including the TUG test (Sebastião et al., 2016), T25w, and vibration threshold measurements from the right and left toe using a Vibratron-II device (Newsome et al., 2011). For the TUG test, the participant was asked to rise from a seated position, walk at their normal pace to a designated line 3 meters from the chair, and return to a seated position. For T25w, the participant was asked to walk 25 feet at their normal pace. The average of two trials was calculated for both the TUG and T25w tests. Participants were then scanned on a 3T Philips Elition X (Philips Medical Systems, Best, The Netherlands) using a dual-channel transmit body coil and 16-channel neurovascular coil for signal reception centered in the cervical SC at C3 to C4 to encompass C2-C5 cervical vertebral levels. The acquisition protocol included:

- Sagittal T2-weighted turbo spin echo (TR/TE = 2500/100 ms, α = 90°, FOV = 160 × 250 mm^2^, 18 slices, voxel size 0.8 × 1 × 2 mm^3^, 2:30 min)
- Multi-echo Fast Field Echo (mFFE) T2*-weighted axial anatomical scan (TR/TE = 700/8 ms, α = 28°, FOV = 160 × 160 mm^2^, 14 slices, voxel size 0.65 × 0.65 × 5 mm^3^, 5:37 min)
- Cardiac-triggered single-shot EPI diffusion sequence (TR = 5 beats (~4000 ms), TE = 77 ms, SENSE (RL) = 1.8, FOV = 80 × 57.5 × 70 mm^3^, 14 slices, resolution = 1.1 × 1.1 × 5 mm^3^, averages = 3, 6:20 min) with a single shell acquisition (15 directions, b = 750 s/mm^2^).

### 2.2 Anatomical image processing

The image processing steps were adapted from prior work and utilized the Spinal Cord Toolbox (SCT) and FSL (Barry et al., 2016; Combes et al., 2022; De Leener et al., 2017; Jenkinson et al., 2012; Smith et al., 2004). Cord segmentations were obtained on sagittal T2 and mFFE scans using *sct_deepseg_sc* (Gros et al., 2019) prior to identification of vertebral levels on sagittal T2, co-registration of the T2 and mFFE images, and creation of GM mask on the mFFE image using *sct_deepseg_gm* (Perone et al., 2018). The WM mask was obtained by subtracting the GM mask from the full cord mask obtained via the initial segmentation. Unsatisfactory segmentations were corrected manually. Lesions were identified and masked by trained neuroradiologists (CM and SS) using ITK-Snap.

Exclusions were made for missing data and data quality, including pwMS with no EDSS scores recorded (n = 2), no sensorimotor testing recorded (n = 2), motion on mFFE images precluding lesion segmentation as judged by trained neuroradiologists CM and SS (n = 2), or without lesion masks (n = 7). After removal of participants, analysis was performed on 46 HCs (31.9 +/- 6.8 years old, 29 females) and 56 pwRRMS (36.5 +/- 7.5 years old, 39 females, EDSS (1 [median], 0-4 [range]).

### 2.3 Diffusion tensor imaging processing

**Fig. 1.**
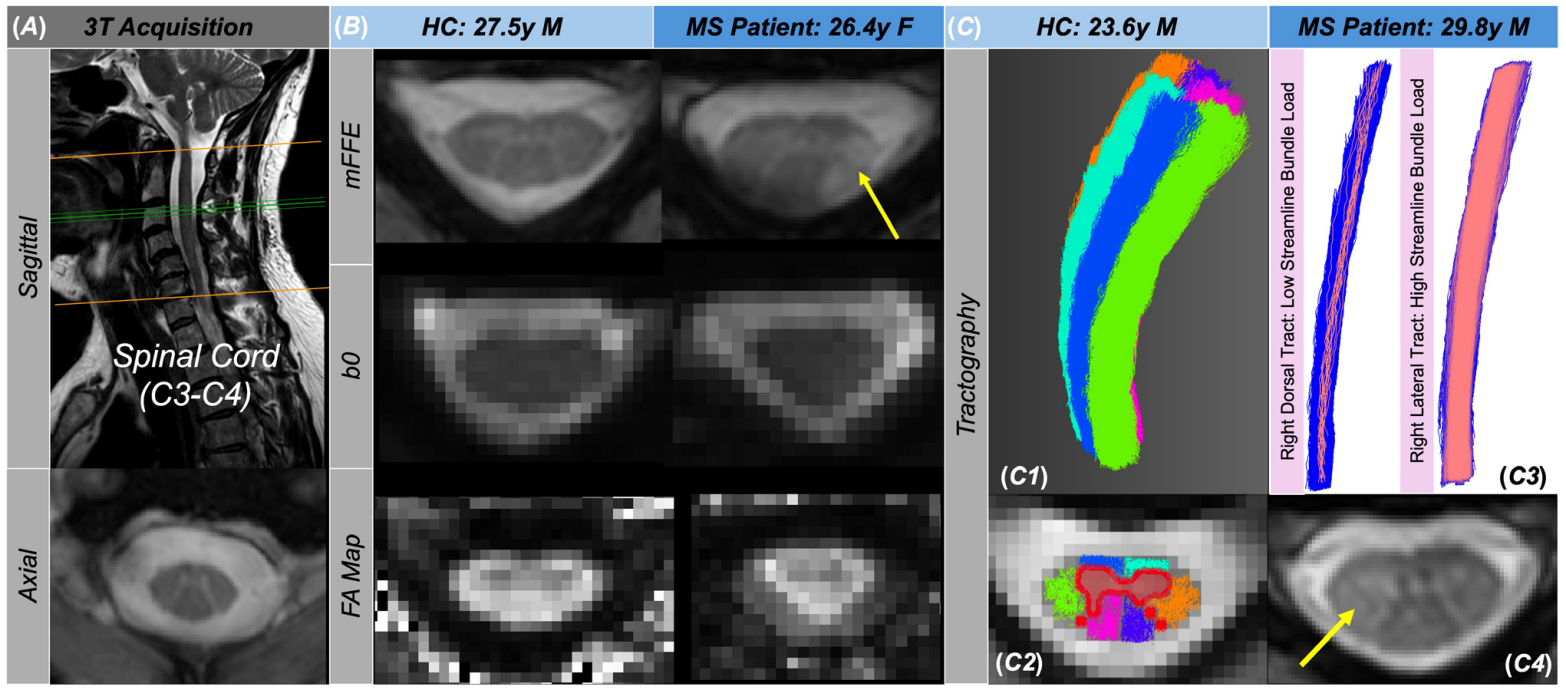
In addition to the single shell diffusion acquisition (15 directions, b = 750 s/mm^2^) centered between cervical levels C3 and C4, the protocol included a sagittal T2-weighted turbo spin echo and mFFE axial anatomical scan (A). The mFFE, diffusion b0, and FA maps are included for a 27.5-year-old HC and 26.4-year-old pwRRMS with a lesion identified via the yellow arrow (B). (C) Tractography is included for a 23.6-year-old HC (*C1*) as visualized via MI-Brain and FiberNavigator. The white matter fiber tracts are split into six bundles (left and right ventral, lateral, and dorsal) with a gray matter mask overlaid on the axial view of the anatomic mFFE image (*C2*). For a 29.8-year-old pwRRMS, two streamline bundles (right dorsal, right lateral) are highlighted in blue, with the streamlines touching lesions highlighted in pink. The right dorsal tract demonstrates low streamline bundle load, while the right lateral tract demonstrates high streamline bundle load (*C3*). The right lateral lesion can be easily visualized on the axial view of the anatomic mFFE (yellow arrow) (*C4*).

Diffusion data underwent denoising via the Marcenko-Pastur PCA algorithm (Cordero-Grande et al., 2019; Tournier et al., 2019; Veraart, Fieremans, et al., 2016; Veraart, Novikov, et al., 2016), Gibbs artifact removal (Kellner et al., 2016; Tournier et al., 2019), and Rician bias noise (Tournier et al., 2019) removal prior to motion correction using *sct_dmri_moco* (Cordero-Grande et al., 2019; Veraart, Fieremans, et al., 2016). Model fitting was performed using SCT to obtain FA, AD, RD, and MD maps (Fig. 1). Atlas images from the PAM50 template, mFFE segmentations, and lesion masks were transformed to the *b* *=* *0* diffusion space via nearest neighbor interpolation warp to prepare for common analysis. Data was quality checked to remove those with significant artifacts.

To derive masks for six WM columns, the PAM50 WM atlas from SCT was used to produce binary masks for dorsal, lateral, and ventral columns in the left and right hemicords, respectively. The signal-to-noise ratio (SNR) was calculated from the *b* *=* *0* image for each participant from the cord-segmented mask (eroded by 1 voxel to prevent spurious contact with the CSF) in the center 5 slices (± 2 slices from the C3-4 intervertebral disk). The volumes of the cord segmentation derived from the mFFE, the six WM binary masks, and the portion of lesion mask segregated to each WM binary mask were calculated using SCT. We also calculated lesion load as the volume of lesions grouped by WM column divided by the volume of the WM column itself. Diffusion-derived microstructural indices, including FA, AD, RD, and MD, were derived and averaged per WM column across all slices (column microstructure).

**Fig. 2.**
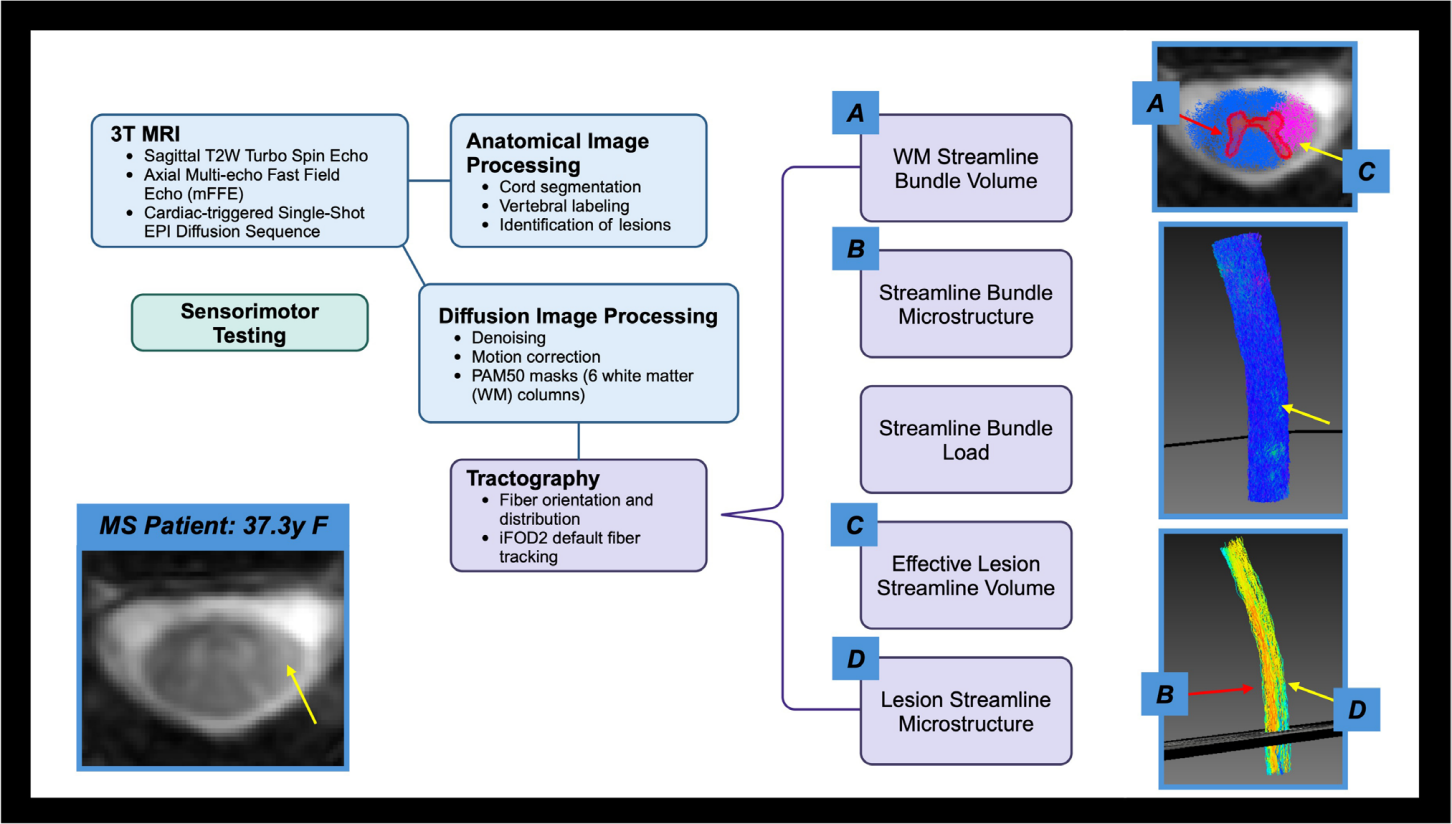
The acquisition and processing pipeline is briefly described. Diffusion data was denoised via the Marcenko-Pastur PCA algorithm, Gibbs, and Rician bias noise removal prior to motion correction. Tractography included response function estimation, calculation of fiber orientation distribution, and the MRtrix fiber tracking algorithm iFOD2 to produce streamlines for each WM bundle. When looking at a lesion (yellow arrow) on an axial slice, the lesion becomes apparent both on anatomical and streamline representations. The tractography measures, both for healthy streamlines (red arrow, A) and streamlines touching lesions (yellow arrow, C), are demonstrated here. Streamlines, as colored by diffusion indices such as FA, can also be separated into healthy streamlines (red arrow, B) and streamlines touching lesions (yellow arrow, D).

**Fig. 3.**
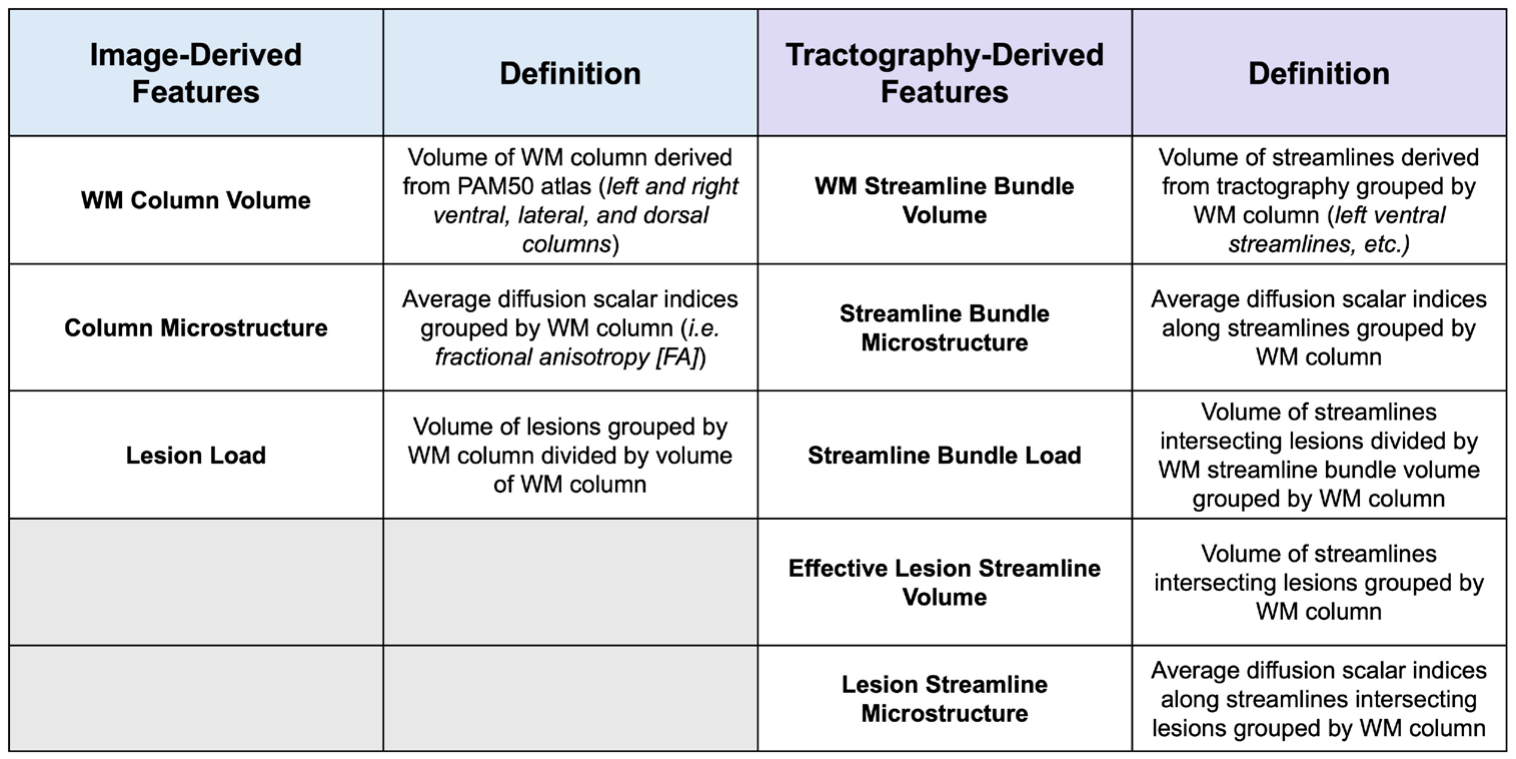
Definitions of column- and streamline-specific WM vocabulary.

For tractography, we estimated the orientations of fibers in each voxel via a response function estimate for WM, GM, and cerebrospinal fluid (CSF) using *dwi2response* from the MRtrix toolbox (Tournier et al., 2019). We then calculated the fiber orientation distribution for all fibers crossing a specific voxel using *dwi2fod*. Based on the response function estimate for GM, WM, and CSF, we then used the fiber tracking algorithm iFOD2 in MRtrix to produce streamlines for each of the six WM fiber tracts (deemed “bundles” for the purpose of tractography) using MRtrix’s *tckgen* with a minimum length requirement of 40 mm seeded from each respective WM mask. Prior to completing this step, we smoothed each WM mask using *mrfilter*, resized the *wmfod* and WM masks to a 1 x 1 x 1 mm^3^ resolution, and binarized the WM masks for voxel values above 0.15 to produce uninterrupted streamlines. The following tractography parameters were calculated for left dorsal (LDo), left ventral (LV), left lateral (LL), right dorsal (RDo), right ventral (RV), and right lateral (RL) bundles: volume of streamlines grouped by WM column (WM streamline bundle volume), volume of streamlines intersecting lesions grouped by WM column (effective lesion streamline volume), and volume of streamlines intersecting lesions divided by WM streamline bundle volume grouped by WM column (streamline bundle load). Diffusion indices, including FA, AD, RD, and MD, were derived using MRtrix as part of “lesionometry” and averaged over the streamlines per WM bundle (streamline bundle microstructure) and the streamlines intersecting lesions per WM bundle (lesion streamline microstructure). Visualization was performed using MI-Brain (Rheault et al., 2016) and FiberNavigator (Chamberland et al., 2014). A representation of tractography in two participants (HC, pwRRMS) is demonstrated in Figure 1. The acquisition and processing pipelines are summarized in Figure 2, with definitions of the tractography vocabulary included in Figure 3.

### 2.4 Statistical analysis

RStudio (v4.4.0, 2024) was used for all statistical analyses (R Core Team, 2024). Demographic and clinical information was compared between HCs and pwRRMS using Welch’s *t*-test for continuous variable, while sex at birth was compared using a chi-squared test. Column microstructure and bundle microstructure were also compared between HCs and pwRRMS using Welch’s *t*-test. Multivariable linear regression was performed to understand if bundle microstructure (dependent variable) is associated with a diagnosis of MS (independent variable), while adjusting for covariates of age, sex, and SNR. These covariates were selected due to know upward bias of diffusion-derived indices at low SNR (Farrell et al., 2007), as well to account for an older and more female MS cohort considering RRMS is a female predominant disease (Coyle, 2021). The same regression was used for column microstructure. For pwRRMS, partial correlations using Spearman’s rank correlation with age, sex, and SNR as covariates were calculated between streamline bundle load for each of the six WM bundles and indices such as EDSS, TUG, T25w, Vibratron threshold left great toe (ability to detect vibration), Vibratron threshold right great toe, and disease duration. The partial correlation coefficient and associated p-values report the strength and significance of the association. Similar analyses were repeated for lesion load on each of the WM columns, and again using lesion streamline microstructure. For all statistical analyses except demographics, FDR correction was performed to account for multiple tests.

## 3 Results

### 3.1 Group comparisons

Demographic data and clinical variables are reported in Table 1. PwRRMS demonstrated significantly increased TUG times and T25w times (p < 0.001, p = 0.003), as well as significantly increased vibration thresholds for the left and right great toes (p < 0.001). The MS cohort was also significantly older than the HC cohort (p < 0.001) with an average age of 36.51 compared to 31.86 in the HC cohort.

**Table 1.** Demographic and clinical averages with standard deviation for HCs and pwRRMS.

	Healthy controls (n = 46)	Multiple sclerosis (n = 56)	T-statistic (T)	Degrees of freedom (df)	p-value (p)
Sex at birth	29F/17M	39F/17M			NS
Age (years)	31.86 +/- 6.78	36.52 +/- 7.45	-3.31	98.92	<0.001**
Disease duration (years)	-	5.72 (0.1 – 20.0)			
EDSS(median [range])	-	1 (0-4)			
TUG (s)	6.10 +/- 0.91	7.29 +/- 1.81	-4.27	84.44	<0.001**
T25w (s)	4.25 +/- 0.62	4.83 +/- 1.17	-3.24	86.50	0.003**
Vibration threshold left	1.30 +/- 0.51	2.13 +/- 1.39	-4.15	71.89	<0.001**
Vibration threshold right	1.23 +/- 0.54	1.95 +/- 1.26	-3.88	77.62	<0.001**

Comparisons were calculated using Welch’s *t*-test, except for sex at birth which was compared using a chi-squared test.

**p < 0.01, NS = not significant.

### 3.2 Column microstructure and streamline microstructure in HCs versus pwRRMS

The average whole cord FA for pwRRMS (mean = 0.58) was significantly lower than HCs (mean = 0.61, p = 0.002; Table 2). The average whole cord AD value was also significantly lower for pwRRMS compared to HCs (p = 0.035; Table 2). Using Welch’s *t-*test, FA was significantly decreased in pwRRMS compared to HCs for the left and right dorsal columns (LDo p = 0.046, RDo p = 0.045; Table 3).

**Table 2.** Comparison of diffusion microstructure derived from whole cord between HCs and pwRRMS via Welch’s *t*-test.

	Whole cord diffusion microstructure: HCs vs. pwRRMS			
	FA	AD (mm^2^/s x 10^-3^)	RD (mm^2^/s x 10^-3^)	MD (mm^2^/s x 10^-3^)
HCs (n = 46)	0.61 +/- 0.03	2.11 +/- 0.11	0.76 +/- 0.09	1.21 +/- 0.09
pwRRMS (n = 56)	0.58 +/- 0.04	2.07 +/- 0.09	0.79 +/- 0.08	1.22 +/- 0.07
p-Value	0.002**	0.035*	0.079	0.710

*p < 0.05, **p < 0.01.

**Table 3.** Comparison of mean FA, AD, RD, and MD, derived along columns and streamlines, between HCs and pwRRMS using Welch’s *t*-test with FDR correction.

Welch’s *t*-test: column and bundle microstructure between HCs and pwRRMS							
Type	Est.	T	df	HC mean	MS mean	p-value	FDR
Column microstructure: FA							
Left dorsal	-0.03	-3.19	99.23	0.63	0.60	0.002	**0.046**
Right dorsal	-0.03	-3.41	99.56	0.61	0.58	0.001	**0.045**
Left lateral	-0.02	-2.28	98.86	0.55	0.53	0.025	0.217
Right lateral	-0.02	-2.66	95.52	0.54	0.52	0.009	0.109
Left ventral	-0.02	-2.06	95.93	0.49	0.47	0.042	0.253
Right ventral	-0.02	-1.85	92.06	0.51	0.49	0.067	0.292
Streamline bundle microstructure: FA							
Left dorsal	-0.01	-0.84	67.63	0.56	0.55	0.403	0.599
Right dorsal	-0.03	-1.70	63.63	0.59	0.56	0.094	0.348
Left lateral	-0.02	-1.86	98.57	0.48	0.45	0.066	0.292
Right lateral	-0.02	-1.27	76.39	0.50	0.48	0.209	0.436
Left ventral	-0.03	-1.80	96.02	0.44	0.41	0.074	0.297
Right ventral	-0.02	-0.99	99.65	0.43	0.41	0.326	0.540
Column microstructure: AD							
Left dorsal	-4.03	-1.30	89.82	2.19	2.15	0.196	0.436
Right dorsal	-3.41	-1.04	93.70	2.19	2.16	0.299	0.537
Left lateral	-8.16	-2.72	90.16	2.31	2.23	0.008	0.109
Right lateral	-4.75	-1.56	91.80	2.33	2.28	0.123	0.414
Left ventral	-7.08	-2.17	97.31	2.38	2.31	0.032	0.221
Right ventral	-6.74	-2.00	98.23	2.35	2.28	0.048	0.258
Streamline bundle microstructure: AD							
Left dorsal	1.48	0.25	59.38	2.22	2.24	0.801	0.906
Right dorsal	0.60	0.10	60.25	2.23	2.23	0.920	0.940
Left lateral	-7.01	-1.38	88.77	2.53	2.46	0.172	0.414
Right lateral	2.50	0.36	69.64	2.34	2.37	0.720	0.906
Left ventral	-7.13	-0.82	97.00	2.45	2.38	0.412	0.599
Right ventral	-14.69	-1.52	99.81	2.52	2.37	0.132	0.414
Column microstructure: RD							
Left dorsal	2.99	1.08	91.23	0.78	0.81	0.285	0.537
Right dorsal	4.09	1.40	98.34	0.82	0.86	0.165	0.414
Left lateral	-1.20	-0.42	89.41	1.01	1.00	0.677	0.879
Right lateral	1.63	0.60	92.46	1.04	1.06	0.550	0.754
Left ventral	-0.83	-0.24	90.84	1.18	1.17	0.809	0.906
Right ventral	-0.60	-0.17	91.78	1.15	1.14	0.868	0.906
Streamline bundle microstructure: RD							
Left dorsal	4.47	1.00	68.69	1.37	1.41	0.319	0.540
Right dorsal	6.23	1.42	68.82	1.33	1.39	0.160	0.414
Left lateral	-1.55	-0.29	85.67	1.74	1.73	0.775	0.906
Right lateral	6.14	1.04	84.40	1.57	1.63	0.302	0.537
Left ventral	-2.19	-0.31	98.02	1.75	1.72	0.754	0.906
Right ventral	-9.58	-1.21	99.59	1.82	1.72	0.231	0.461
Column microstructure: MD							
Left dorsal	0.65	0.24	89.23	1.25	1.25	0.812	0.906
Right dorsal	1.59	0.55	96.64	1.27	1.29	0.583	0.777
Left lateral	-3.52	-1.27	88.17	1.44	1.41	0.209	0.436
Right lateral	-0.50	-0.18	92.00	1.47	1.47	0.855	0.906
Left ventral	-2.91	-0.91	92.93	1.58	1.55	0.365	0.566
Right ventral	-2.65	-0.78	94.44	1.55	1.52	0.436	0.616
Streamline bundle microstructure: MD							
Left dorsal	5.96	1.42	78.94	0.94	1.00	0.160	0.414
Right dorsal	9.04	2.25	78.04	0.89	0.98	0.027	0.217
Left lateral	1.19	0.21	86.58	1.35	1.36	0.837	0.906
Right lateral	7.96	1.40	92.11	1.18	1.26	0.164	0.414
Left ventral	0.27	0.04	98.66	1.40	1.40	0.966	0.966
Right ventral	-7.03	-0.96	99.29	1.46	1.39	0.341	0.545

For AD, MD, and RD, estimates are reported as 10^-5^ and mean values are reported as 10^-3^.

FDR p-values are bolded if p < 0.05.

T = T-statistic, df = degrees of freedom.

### 3.3 Relationship between diffusion microstructure and demographics in HCs and pwRRMS

**Fig. 4.**
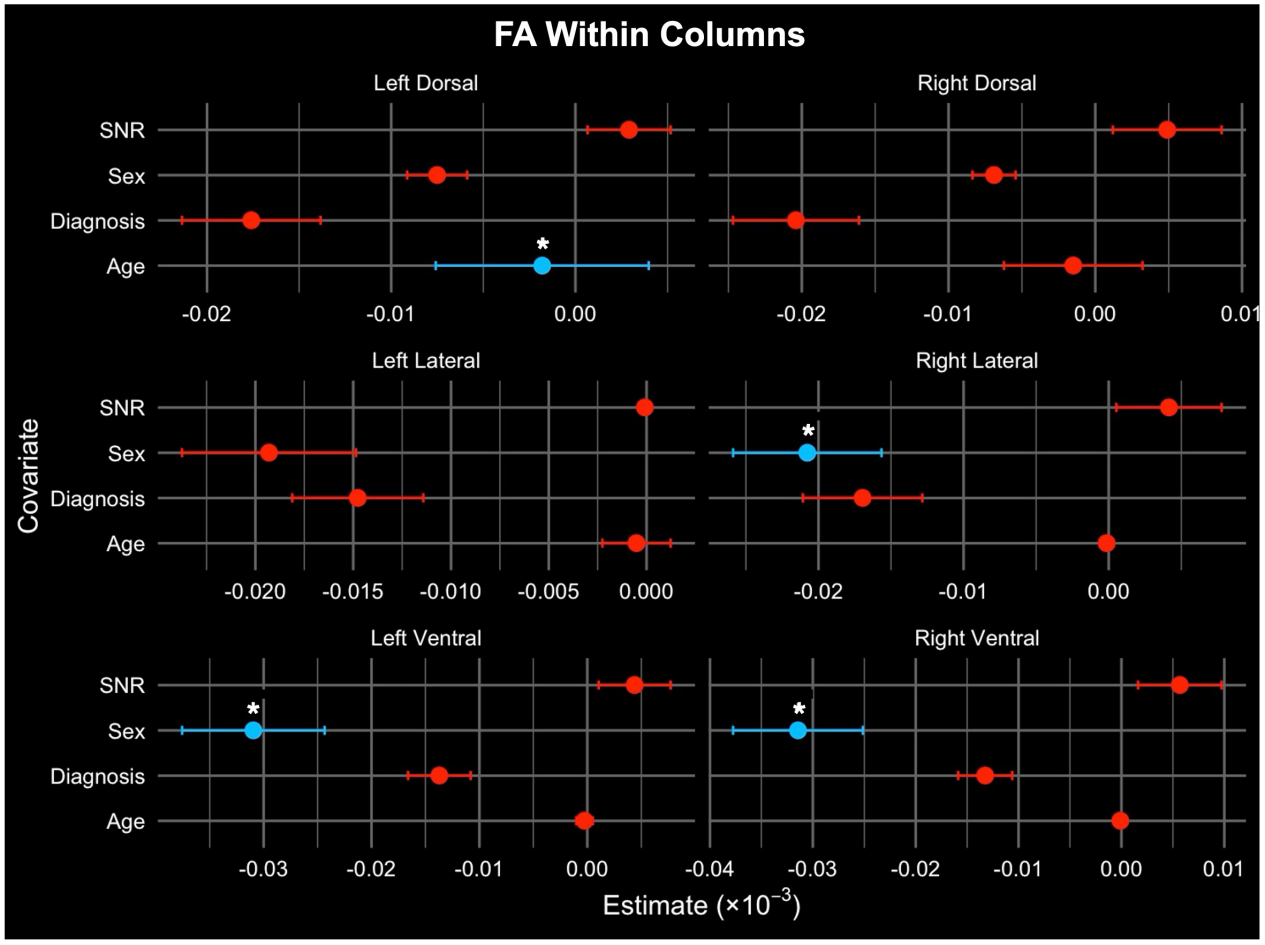
Comparison of average column-wise FA per six WM bundles and variables such as diagnosis, age, sex, and SNR using multivariable linear regression results. The x-axis refers to the regression coefficients with the change in dependent variable for a one-unit change in the corresponding covariate. Significant relationships are denoted with a “*” (FDR corrected p < 0.05).

**Fig. 5.**
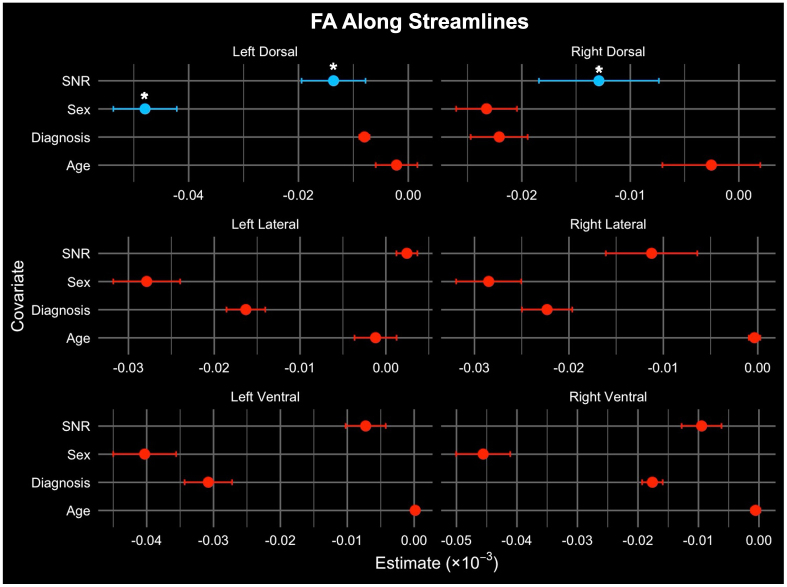
Comparison of average FA derived along streamlines per six WM bundles and variables such as diagnosis, age, sex, and SNR using multivariable linear regression results. The x-axis refers to the regression coefficients with the change in dependent variable for a one-unit change in the corresponding covariate. Significant relationships are denoted with a “*” (FDR corrected p < 0.05).

Linear regression was used to model the association of age, sex, SNR, and diagnosis (HC or pwRRMS) with WM column microstructure. Column FA was significantly higher with younger age (LDo p = 0.016) and the male sex (RL p = 0.041, LV p = 0.004, RV p = 0.009; Table 4). Streamline bundle FA was significantly higher with the male sex (LDo p = 0.016) and decreased SNR (LDo p = 0.016, RDo p = 0.024; Table 4). After FDR correction, there were no remaining significant associations between either column or streamline FA and diagnosis status. Visualization of these results are included in Figures 4 and 5.

**Table 4.** Multiple variable linear regression of FA with diagnosis (HC vs. MS) as the variable of interest and age, sex, and SNR as covariates.

Multiple variable linear regression: FA				
Name	Estimate	T	p-value	FDR
Left dorsal column				
Age	-1.80	-2.95	0.004	**0.016**
Sex	-7.50	-0.83	0.409	0.584
SNR	2.92	1.15	0.254	0.414
Diagnosis	-17.59	-1.92	0.057	0.141
Right dorsal column				
Age	-1.49	-2.41	0.018	0.061
Sex	-6.89	-0.75	0.456	0.635
SNR	4.91	1.89	0.061	0.148
Diagnosis	-20.39	-2.19	0.031	0.095
Left lateral column				
Age	-0.51	-0.89	0.377	0.564
Sex	-19.30	-2.27	0.026	0.083
SNR	-0.08	-0.03	0.974	0.986
Diagnosis	-14.76	-1.71	0.090	0.193
Right lateral column				
Age	-0.13	-0.24	0.810	0.900
Sex	-20.77	-2.61	0.011	**0.041**
SNR	4.16	1.85	0.067	0.159
Diagnosis	-16.95	-2.10	0.038	0.113
Left ventral column				
Age	-0.24	-0.39	0.698	0.838
Sex	-30.95	-3.37	0.001	**0.004**
SNR	4.40	1.70	0.092	0.195
Diagnosis	-13.70	-1.48	0.143	0.275
Right ventral column				
Age	-0.09	-0.13	0.896	0.954
Sex	-31.44	-3.22	0.002	**0.009**
SNR	5.69	2.07	0.041	0.119
Diagnosis	-13.23	-1.34	0.183	0.323
Left dorsal streamlines				
Age	-2.11	-1.93	0.057	0.141
Sex	-47.94	-2.95	0.004	**0.016**
SNR	-13.60	-2.97	0.004	**0.016**
Diagnosis	-7.96	-0.48	0.630	0.775
Right dorsal streamlines				
Age	-2.52	-2.30	0.023	0.077
Sex	-23.24	-1.43	0.156	0.290
SNR	-12.89	-2.82	0.006	**0.024**
Diagnosis	-22.07	-1.34	0.183	0.323
Left lateral streamlines				
Age	-1.18	-1.25	0.216	0.363
Sex	-27.86	-1.99	0.050	0.129
SNR	2.47	0.62	0.534	0.704
Diagnosis	-16.30	-1.15	0.254	0.414
Right lateral streamlines				
Age	-0.33	-0.31	0.760	0.873
Sex	-28.48	-1.76	0.081	0.180
SNR	-11.23	-2.47	0.015	0.053
Diagnosis	-22.30	-1.36	0.176	0.315
Left ventral streamlines				
Age	0.18	0.16	0.870	0.941
Sex	-40.28	-2.40	0.018	0.061
SNR	-7.23	-1.53	0.129	0.258
Diagnosis	-30.76	-1.81	0.073	0.170
Right ventral streamlines				
Age	-0.53	-0.39	0.694	0.837
Sex	-45.56	-2.28	0.025	0.081
SNR	-9.46	-1.68	0.095	0.200
Diagnosis	-17.59	-0.87	0.386	0.565

The values in the estimate column are 10^-3^ and df = 97.

FDR p-values are bolded if p < 0.05.

The same analysis was repeated for column microstructure indices AD, RD, and MD, as well as streamline microstructure indices AD (Table 5 in Supplemental Materials), RD (Table 6 in Supplemental Materials), and MD (Table 7 in Supplemental Materials) included in the supplemental materials. Increased AD was associated with younger age (RV column, p = 0.038) and decreased SNR (LDo streamlines p = 0.044, LV streamlines p = 0.004, RV streamlines p = 0.035). Decreased RD was associated with the male sex (RL column p = 0.044, LV column p = 0.004, RV column p = 0.004) and increased SNR (LV streamlines p = 0.004). Decreased MD was associated with the male sex (LV column p = 0.016, RV column p = 0.013) and increased SNR (LV streamlines p = 0.004).

### 3.4 Streamline bundle load and lesion load versus clinical variables in pwRRMS

Partial Spearman’s correlations, considering covariates of age, sex, and SNR, demonstrated significant uncorrected associations between streamline or lesion load and clinical variables in pwRRMS. However, these associations did not survive FDR correction (Table 8 in Supplemental Materials). Additionally, the expected association between altered ventral columns and mobility (TUG, T25w) and altered dorsal columns and vibration (Vibratron Threshold Left, Vibratron Threshold Right) was not observed in this analysis.

### 3.5 Streamline microstructure and lesion streamline microstructure versus clinical variables in pwRRMS

Like the previous analysis, partial correlations using Spearman’s correlation, considering covariates like age, sex, and SNR, demonstrated significant uncorrected associations between clinical variables and streamline FA or lesion streamline FA in pwRRMS that did not survive FDR correction. There were no noted associations with EDSS, Vibratron Threshold Right, or Vibratron Threshold Left (Table 9 in Supplemental Materials).

## 4 Discussion

Given tractography applied in the MS cervical SC is relatively novel, we sought to derive WM streamlines according to the iFOD2 algorithm and relate streamline diffusion-derived indices to clinical measures and lesion load. We found associations between reduced FA within WM columns and age and sex, with limited expansion to WM streamlines. We did not determine significant relationships between clinical variables and either lesion load or diffusion-derived indices.

### 4.1 PwRRMS demonstrate greater disability compared to HCs

As expected according to existing knowledge of clinical impairment of pwRRMS, our MS cohort demonstrated significantly increased TUG, T25w, vibration threshold for the left toe, and vibration threshold for the right toe compared to HCs (Cohen et al., 2014; Sebastião et al., 2016).

### 4.2 Column microstructure captures differences between pwRRMS and HCs, while streamline microstructure does not

From our work, spatially segregated analysis of DTI indices may provide additional details to determine which regions of the SC are most impacted in pwRRMS. Our segregated column analysis aligned with the patterns seen at the whole-cord level, including decreased FA in pwRRMS versus HCs, lending merit to our WM column parcellation.

The decreased FA in pwRRMS compared to HCs has been noted prior—including reduced mean SC FA in pwRRMS and diminishing FA with longitudinal follow-up visits—indicating a possible loss of fiber coherence or damage to tissue microstructure in MS cohorts (Agosta et al., 2008; Combes et al., 2022; Valsasina et al., 2005). Furthermore, in the study using PICo and probabilistic tractography connectivity measurements, pwMS demonstrated reduced voxel-based connectivity, FA, and total *N*-acetyl-aspartate from ^1^H-MR spectroscopy in the lateral corticospinal and posterior tracts of the cervical SC compared to HCs (Ciccarelli et al., 2007).

We found significantly different dorsal column FA between pwRRMS and HCs, with no differences in streamline microstructure. When applying automated WM bundle segmentation techniques such as TractSeg in traumatic brain injury patients, Tallus et al. found decreased FA and MD compared to deterministic DTI techniques, though they recognized tractography methods are not designed to be comparable to other DTI techniques given how they are defined (Schilling et al., 2021; Tallus et al., 2023). Other groups have demonstrated decreased FA in normal-appearing WM split into lateral, posterior, and central SC regions, similar to how we have defined them in this study, as well as decreased FA in whole cord normal-appearing WM in pwRRMS versus HCs (Combes et al., 2022; Hesseltine et al., 2006). Thus, our determination of significantly lower FA in pwRRMS when derived from columns aligns with previous findings, and perhaps suggests that additional streamline parcellation is not necessary to extract meaningful differences between pwMS and controls. Even so, while deriving DTI microstructure within WM columns instead of averaging over the whole cord may provide additional value, measures of tractography may still be important to visualize damage across not otherwise captured by conventional imaging.

### 4.3 Females and older participants demonstrate reduced WM integrity

Our study provides evidence for age- and sex-related differences in the cervical SC, in a cohort of substantial size, with column microstructure demonstrating greater sensitivity compared to streamline microstructure.

In the linear regression models, females demonstrated reduced WM integrity compared to their male counterparts as captured by reduced FA, increased RD, and increased MD in WM columns. This finding was mainly localized to lateral and ventral columns versus streamlines, with only one observation surviving FDR correction in streamlines (FA LDo streamlines). Females have been shown to have decreased global FA and increased MD in brain regions such as the anterior cingulum compared to males, though there are mixed findings of related sex-effects in the SC (Matijevic & Ryan, 2021; Saksena et al., 2016). Furthermore, a study employing advanced WM microstructure techniques in the brain, such as neurite orientation dispersion and density imaging (NODDI), revealed that females exhibit greater WM dispersion and decreased anisotropy compared to males but that sex-related diffusivity differences are dependent on brain region (Lawrence et al., 2021). Our findings contribute to the growing body of evidence suggesting sex differences in the SC may exist but have yet to be fully elucidated.

Similarly, our regression analysis revealed significant associations between reduced WM integrity—as represented by reduced FA and AD—and older age. These findings were localized to WM columns, but did not extend to WM streamlines. FA and RD have been found to decrease and increase, respectively, with age in cerebral WM, and we show similar FA findings in the cervical SC suggestive of reduced WM integrity with age (Behler et al., 2021; Lindberg et al., 2010; Mamata et al., 2005; Matijevic & Ryan, 2021; Zahr et al., 2009). AD has been shown to both increase and decrease with age in the cerebral WM, and age-related decreases in AD may interpreted as disruption in fiber tract organization (Bennett et al., 2010). Even with this observation, it is important to note our study focused on a relatively young population (31.86 years old for HCs, 36.52 years old for pwRRMS) and thus age-related changes are likely minimal compared to studies performed in older populations.

Our inclusion of SNR as a covariate served to account for the known upward bias of FA at low SNR, with an average SNR of 11.16 (HC) and 10.71 (pwRRMS) deemed insignificantly different to cause the observed differences between cohorts (Farrell et al., 2007). Even so, it is interesting to consider the significant associations between decreased SNR and increased streamline microstructure, knowing that low SNR indicates diminished visual contrast and may threaten the accuracy of DTI-derived microstructural indices. In a small area like the SC, SNR may be a limiting factor for tractography, especially when compared to a region like the brain with greater SNR and demonstrated tractography benefits.

### 4.4 Streamline bundle load and microstructure do not demonstrate significant associations with clinical variables

We sought to explore how streamlines associated with lesions relate to clinical outcomes, such as EDSS, TUG, and T25w, compared to overall column-wise lesion load. However, there was no trend favoring either streamline bundle load or lesion load to best correlate with clinical variables, including no significant associations with measures like EDSS, TUG, and T25w. EDSS has been found to be independently associated with SC lesion load in relapsing-remitting and progressive MS populations, though it is worth noting our inclusion of only low to mild-disability pwMS with an EDSS of less than 4 compared to 6 in other relapsing-remitting populations studied (Kearney et al., 2015). Additionally, we did not utilize the 9-hole peg test (9HPT) to capture upper-limb function, though the PICO study showed significant correlations between posterior column voxel-based connectivity and 9HPT scores suggestive of diminished connectivity with greater upper limb dysfunction (Ciccarelli et al., 2007).

We also investigated associations between FA and clinical variables, focusing on microstructure derived from streamlines and lesion streamlines within WM bundles. While there were trends favoring decreased FA with increased TUG and T25w times for both streamlines and lesion-specific streamlines within WM bundles, these associations were not significant following FDR correction. It is known that traditional structural imaging does not always correlate well with clinical findings, whereas diffusion FA can serve as a correlate for motor function, particularly in participants with SC injury (Chang et al., 2010). Associations between SC DTI microstructure and specific motor or sensory tasks in pwRRMS are limited (Klineova et al., 2016), though there is evidence of decreased posterior column FA and increased RD with worse ambulation and vibration sensation in participants with demyelinating disease (Naismith et al., 2013). Our results suggest that anatomic parcellation of columns or WM tracts outside of specific fiber pathways may not provide additional clinically relevant information in the SC, beyond what has already been established from whole-cord or GM specific analyses. There may be utility in revisiting our analysis to include specific WM fiber tracts, rather than general regions of the cervical SC.

### 4.5 Limitations

Limitations in the current study involve both our recruited MS population as well as our method of tractogram creation. There was also a significant difference in the age of our cohorts, with pwMS being on average ~5 years older than our HC cohort. It has been suggested that changes in the pathophysiology of MS may be related to age, albeit with marginal differences over the span of 5 years (Wang et al., 2014), and thus we accounted for any significant age-related effects by including age as a covariate in all statistical analyses (Macaron et al., 2023). Additionally, our MS population consisted of pwMS with relatively low disability (EDSS less than or equal to 4), possibly accounting for our lack of significant associations with EDSS throughout our analysis. For future analysis, the tractogram pipeline should be applied to an MS population with more severe disability to determine if the trends noted herein remain consistent. Furthermore, partial volume effects must be considered in light of the small size of the SC with limited spatial resolution, as our results suggest that SNR does play a role in streamline-derived diffusion indices. Ultra-high field MRI, such as 7T, may potentially improve the SNR and help mitigate significant noise to best detect and characterize WM microscopic changes (Gatto et al., 2018).

For streamline creation, we elected to use the default fiber tracking algorithm iFOD2 in MRtrix, selected for streamlines with at least a minimum length of 40 mm, and elected to seed streamlines based on the 15% threshold of each WM mask derived from the PAM50 template. These options were selected after multiple iterations and qualitative assessment, though we did not perform any quantitative assessment based on these streamline parameters. It is possible that further quantitative evaluation of possible parameter combinations could produce more robust streamline tracking, though we feel confident in our parameter selection as we thoroughly tested different tracking algorithms such as SD_STREAM, minimum lengths between 30 and 60 mm, and threshold values between 0 and 100%. Additionally, the 15% threshold expanded our WM mask outside of just the WM, and thus our streamline and effective lesion streamline volumes included streamlines outside of just the WM masks compared to lesion volume confined to the WM masks. However, given seeding from WM masks allows for meandering streamlines but does not require streamlines to fill the entire seeded volume, we feel this 15% threshold was still appropriate; furthermore, we normalized effective lesion streamline volumes and lesion volumes via creation of streamline bundle load and lesion load, respectively, taking into account the volume of the WM region depending on if the calculation was streamline or cord-based.

Pre-processing in SC diffusion is still an open area of investigation. It should be noted that Gibbs ringing artifact removal via MRtrix relies on the method proposed by Kellner et al. using local subvoxel-shifts (Kellner et al., 2016). This method is designed to work with images acquired with full k-space coverage. While it is still relatively robust in the presence of partial Fourier, it may be more appropriate to use the method proposed by Lee et al. where the conventional method is applied twice to minimize ringing patterns of different periods (Lee et al., 2021).

Despite tractometry in the brain of pwMS contributing valuable information to explain task-specific performance, results in the SC are relatively inconclusive. Chamberland et al. determined tract-based indices offer greater sensitivity to assess working memory and verbal fluency deficits in pwMS compared to traditional lesion load analysis (Chamberland et al., 2021). Though we established the ability to reliably model streamlines in the SC, perhaps generation of streamlines is not necessary in the SC so long as WM columns can be visualized and segmented on high-resolution MR images. We hope this analysis serves to add to the tractography literature and look forward to future work applying tractography in the SC.

## 5 Conclusion

We applied diffusion tractography in the MS SC, separated into six distinct WM regions, to disentangle whether whole cord, column-based, or streamline-based analysis provides the greatest clinical value for pwRRMS. Analysis based on distinct WM columns may, indeed, provide a more nuanced understanding of how diffusion indices change with age and sex compared to traditional whole cord techniques, while more complicated tractography may be limited in the relevant information it can provide outside of column analysis. This was also noted when parsing out streamline bundle load versus lesion load associations with clinical variables. We found that females and older participants demonstrate reduced WM integrity compared to their counterparts, highlighted by DTI microstructure such as decreased FA. Decreased FA, calculated along streamlines and lesion streamlines within WM bundles, was associated with increased TUG and T25w times, signifying diminished motor function may be related to diminished cord anisotropy and increased SC disorganization. However, these findings did not survive FDR correction. In future studies, we would like to apply this analysis to pwRRMS with more severe disability and explore other fiber tracking algorithms more in depth.

## Supplementary Material

Supplementary Material

## Data Availability

All data generated or analyzed during the study are included in methods section of this paper. We welcome collaboration and access to our de-identified imaging and sensorimotor data as well as the code written to analyze it. To abide by local institutional policies, a signed data transfer agreement will be required for access and download.
