# Supplementary material for: Tract-specific analysis of diffusion MRI at 3T detects cervical spinal cord aberrations in multiple sclerosis
Source: Imaging Neurosci (Camb). 2025 Jul 7;3:IMAG.a.72. doi: 10.1162/IMAG.a.72 (PMC12330836; doi:10.1162/IMAG.a.72)
Supplement: Supplementary Material [file IMAG.a.72_supp.pdf]

Multiple Variable Linear Regression: AD

Name	*Estimate*	*T*	*P-Value*	*FDR*
Left Dorsal Column				
Age	0.13	0.60	0.552	0.712
Sex	-2.01	-0.62	0.540	0.704
SNR	0.23	0.25	0.807	0.900
Diagnosis	-4.41	-1.33	0.186	0.326
Right Dorsal Column				
Age	0.08	0.32	0.746	0.873
Sex	-1.99	-0.57	0.569	0.723
SNR	-0.61	-0.63	0.533	0.704
Diagnosis	-3.91	-1.11	0.270	0.432
Left Lateral Column				
Age	-0.18	-0.85	0.395	0.571
Sex	2.73	0.88	0.383	0.564
SNR	1.69	1.93	0.056	0.141
Diagnosis	-6.74	-2.14	0.035	0.105
Right Lateral Column				
Age	-0.24	-1.10	0.273	0.434
Sex	4.42	1.39	0.166	0.306
SNR	-0.94	-1.06	0.292	0.452
Diagnosis	-4.37	-1.36	0.176	0.315
Left Ventral Column				
Age	-0.44	-1.92	0.058	0.142
Sex	6.22	1.83	0.070	0.165
SNR	0.28	0.29	0.769	0.879
Diagnosis	-5.32	-1.55	0.125	0.254
Right Ventral Column				
Age	-0.60	-2.63	0.010	**0.038**
Sex	6.91	2.02	0.046	0.125
SNR	-0.90	-0.93	0.353	0.533
Diagnosis	-4.78	-1.38	0.171	0.311
Left Dorsal Streamlines				
Age	-0.21	-0.55	0.584	0.734
Sex	-0.31	-0.06	0.956	0.978
SNR	-4.05	-2.55	0.012	**0.044**
Diagnosis	0.65	0.11	0.910	0.958
Right Dorsal Streamlines				
Age	-0.09	-0.22	0.824	0.903
Sex	-3.42	-0.58	0.563	0.719
SNR	-3.41	-2.06	0.042	0.119
Diagnosis	-0.29	-0.05	0.961	0.978
Left Lateral Streamlines				
Age	-0.01	-0.02	0.986	0.994
Sex	6.06	1.13	0.260	0.419
SNR	1.11	0.74	0.461	0.636

Diagnosis	-6.88	-1.27	0.207	0.354
Right Lateral Streamlines				
Age	-0.81	-1.77	0.080	0.179
Sex	-0.53	-0.08	0.938	0.977
SNR	-4.15	-2.18	0.032	0.097
Diagnosis	4.43	0.65	0.520	0.704
Left Ventral Streamlines				
Age	-1.14	-1.99	0.049	0.128
Sex	-4.18	-0.49	0.625	0.773
SNR	-8.39	-3.50	0.001	**0.004**
Diagnosis	-5.30	-0.61	0.540	0.704
Right Ventral Streamlines				
Age	-1.37	-2.05	0.043	0.120
Sex	-3.17	-0.32	0.750	0.873
SNR	-7.45	-2.68	0.009	**0.035**
Diagnosis	-11.45	-1.14	0.255	0.414

Table 5: Multiple variable linear regression of AD with diagnosis (HC vs. MS) as the variable of interest and age, sex, and SNR as covariates. The values in the estimate column are 10^{-5} and df = 97. FDR p-values are bolded if $p < 0.05$.

Multiple Variable Linear Regression: RD

Name	*Estimate*	*T*	*P-Value*	*FDR*
Left Dorsal Column				
Age	0.47	2.42	0.017	0.059
Sex	0.99	0.35	0.730	0.867
SNR	-0.21	-0.26	0.795	0.896
Diagnosis	0.65	0.22	0.824	0.903
Right Dorsal Column				
Age	0.37	1.78	0.078	0.178
Sex	0.64	0.21	0.836	0.908
SNR	-1.31	-1.50	0.137	0.267
Diagnosis	1.73	0.55	0.582	0.734
Left Lateral Column				
Age	0.03	0.14	0.892	0.954
Sex	6.13	2.07	0.041	0.119
SNR	0.90	1.09	0.280	0.442
Diagnosis	-1.32	-0.44	0.660	0.800
Right Lateral Column				
Age	-0.06	-0.31	0.754	0.873
Sex	7.10	2.57	0.012	**0.044**
SNR	-1.23	-1.59	0.115	0.240

Diagnosis	0.88	0.31	0.754	0.873
Left Ventral Column				
Age	-0.20	-0.88	0.383	0.564
Sex	11.14	3.27	0.001	**0.004**
SNR	-1.16	-1.21	0.230	0.381
Diagnosis	-1.15	-0.33	0.739	0.873
Right Ventral Column				
Age	-0.37	-1.55	0.124	0.254
Sex	11.52	3.27	0.001	**0.004**
SNR	-2.00	-2.01	0.047	0.127
Diagnosis	-0.55	-0.15	0.879	0.946
Left Dorsal Streamlines				
Age	0.08	0.28	0.780	0.887
Sex	6.35	1.43	0.155	0.290
SNR	-2.15	-1.73	0.088	0.190
Diagnosis	2.69	0.60	0.551	0.712
Right Dorsal Streamlines				
Age	0.25	0.85	0.398	0.572
Sex	0.28	0.06	0.951	0.978
SNR	-1.73	-1.39	0.169	0.310
Diagnosis	4.25	0.95	0.346	0.526
Left Lateral Streamlines				
Age	0.17	0.44	0.659	0.800
Sex	9.90	1.77	0.080	0.179
SNR	0.38	0.24	0.810	0.900
Diagnosis	-2.80	-0.50	0.621	0.772
Right Lateral Streamlines				
Age	-0.60	-1.49	0.139	0.269
Sex	3.76	0.62	0.534	0.704
SNR	-2.58	-1.52	0.132	0.260
Diagnosis	7.56	1.24	0.218	0.363
Left Ventral Streamlines				
Age	-0.96	-2.06	0.042	0.119
Sex	0.49	0.07	0.944	0.977
SNR	-6.74	-3.48	0.001	**0.004**
Diagnosis	-0.79	-0.11	0.910	0.958
Right Ventral Streamlines				
Age	-1.07	-1.94	0.055	0.140
Sex	2.24	0.27	0.785	0.889
SNR	-5.68	-2.47	0.015	0.053
Diagnosis	-7.31	-0.88	0.379	0.564

Table 6: Multiple variable linear regression of RD with diagnosis (HC vs. MS) as the variable of interest and age, sex, and SNR as covariates. The values in the estimate column are 10^{-5} and df = 97. FDR p-values are bolded if $p < 0.05$.

Multiple Variable Linear Regression: MD

Name	*Estimate*	*T*	*P-Value*	*FDR*
Left Dorsal Column				
Age	0.35	1.86	0.065	0.156
Sex	-0.01	0.00	0.997	0.997
SNR	-0.06	-0.08	0.936	0.977
Diagnosis	-1.04	-0.36	0.718	0.857
Right Dorsal Column				
Age	0.27	1.32	0.190	0.330
Sex	-0.23	-0.08	0.940	0.977
SNR	-1.08	-1.24	0.216	0.363
Diagnosis	-0.15	-0.05	0.962	0.978
Left Lateral Column				
Age	-0.04	-0.22	0.829	0.904
Sex	4.99	1.74	0.085	0.185
SNR	1.17	1.44	0.152	0.287
Diagnosis	-3.13	-1.08	0.284	0.444
Right Lateral Column				
Age	-0.12	-0.63	0.531	0.704
Sex	6.21	2.24	0.028	0.087
SNR	-1.14	-1.46	0.148	0.282
Diagnosis	-0.87	-0.31	0.757	0.873
Left Ventral Column				
Age	-0.28	-1.29	0.201	0.347
Sex	9.50	2.94	0.004	**0.016**
SNR	-0.68	-0.75	0.458	0.635
Diagnosis	-2.54	-0.78	0.439	0.620
Right Ventral Column				
Age	-0.45	-1.99	0.049	0.128
Sex	9.98	3.00	0.003	**0.013**
SNR	-1.63	-1.74	0.085	0.185
Diagnosis	-1.96	-0.58	0.563	0.719
Left Dorsal Streamlines				
Age	0.23	0.81	0.423	0.601
Sex	9.68	2.29	0.024	0.079
SNR	-1.21	-1.01	0.315	0.485
Diagnosis	3.71	0.86	0.389	0.566
Right Dorsal Streamlines				
Age	0.42	1.53	0.129	0.258
Sex	2.12	0.52	0.607	0.759
SNR	-0.89	-0.77	0.443	0.622
Diagnosis	6.52	1.57	0.121	0.250
Left Lateral Streamlines				
Age	0.25	0.63	0.527	0.704
Sex	11.82	2.00	0.049	0.128
SNR	0.01	0.01	0.994	0.997

Diagnosis	-0.77	-0.13	0.898	0.954
Right Lateral Streamlines				
Age	-0.50	-1.27	0.208	0.354
Sex	5.90	1.00	0.320	0.489
SNR	-1.79	-1.08	0.285	0.444
Diagnosis	9.12	1.53	0.130	0.258
Left Ventral Streamlines				
Age	-0.86	-2.04	0.045	0.124
Sex	2.82	0.45	0.655	0.800
SNR	-5.92	-3.33	0.001	**0.004**
Diagnosis	1.46	0.23	0.820	0.903
Right Ventral Streamlines				
Age	-0.92	-1.80	0.075	0.173
Sex	4.94	0.65	0.516	0.704
SNR	-4.80	-2.25	0.027	0.085
Diagnosis	-5.23	-0.68	0.497	0.682

Table 7: Multiple variable linear regression of MD with diagnosis (HC vs. MS) as the variable of interest and age, sex, and SNR as covariates. The values in the estimate column are 10^{-5} and df = 97. FDR p-values are bolded if $p < 0.05$.

Spearman's Partial Correlation:
Streamline Bundle Load and Lesion Load vs. Clinical Metrics

		**EDSS**					
		Left Dorsal	Right Dorsal	Left Lateral	Right Lateral	Left Ventral	Right Ventral
Streamline Bun. Load	Rho	0.056	0.037	0.133	0.136	0.154	0.429
	P Val.	0.711	0.804	0.383	0.358	0.384	0.010
	FDR	0.897	0.898	0.801	0.801	0.801	0.182
Lesion Load	Rho	0.076	-0.014	0.103	0.036	0.029	0.143
	P Val.	0.625	0.928	0.51	0.811	0.869	0.398
	FDR	0.878	0.968	0.801	0.898	0.921	0.801
		**TUG**					
		Left Dorsal	Right Dorsal	Left Lateral	Right Lateral	Left Ventral	Right Ventral
Streamline Bun. Load	Rho	-0.171	0.055	0.127	0.070	0.036	-0.148
	P Val.	0.252	0.712	0.405	0.638	0.841	0.395
	FDR	0.801	0.897	0.801	0.878	0.918	0.801
Lesion Load	Rho	0.066	0.070	0.109	0.003	0.099	-0.178
	P Val.	0.672	0.641	0.485	0.982	0.564	0.293
	FDR	0.896	0.878	0.801	0.982	0.864	0.801
		**T25w**					

		Left Dorsal	Right Dorsal	Left Lateral	Right Lateral	Left Ventral	Right Ventral
Streamline Bun. Load	Rho	-0.047	0.118	0.279	0.126	0.046	-0.064
	P Val.	0.755	0.424	0.064	0.392	0.798	0.715
	FDR	0.897	0.801	0.647	0.801	0.898	0.897
Lesion Load	Rho	0.115	0.119	0.277	0.046	0.170	-0.119
	P Val.	0.457	0.426	0.073	0.762	0.321	0.482
	FDR	0.801	0.801	0.647	0.897	0.801	0.801
		**Vibratron Threshold Left**					
		Left Dorsal	Right Dorsal	Left Lateral	Right Lateral	Left Ventral	Right Ventral
Streamline Bun. Load	Rho	0.105	0.097	0.279	0.224	0.013	0.128
	P Val.	0.483	0.512	0.063	0.126	0.942	0.463
	FDR	0.801	0.801	0.647	0.647	0.968	0.801
Lesion Load	Rho	0.160	0.079	0.258	0.231	0.161	0.117
	P Val.	0.301	0.597	0.095	0.119	0.349	0.492
	FDR	0.801	0.877	0.647	0.647	0.801	0.801
		**Vibratron Threshold Right**					
		Left Dorsal	Right Dorsal	Left Lateral	Right Lateral	Left Ventral	Right Ventral
Streamline Bun. Load	Rho	0.025	-0.082	0.140	0.110	0.043	0.061
	P Val.	0.870	0.579	0.361	0.456	0.809	0.728
	FDR	0.921	0.869	0.801	0.801	0.898	0.897
Lesion Load	Rho	0.147	0.049	0.120	0.069	0.125	-0.007
	P Val.	0.342	0.742	0.443	0.647	0.466	0.966
	FDR	0.801	0.897	0.801	0.878	0.801	0.979
		**Disease Duration**					
		Left Dorsal	Right Dorsal	Left Lateral	Right Lateral	Left Ventral	Right Ventral
Streamline Bun. Load	Rho	-0.301	-0.174	-0.384	-0.207	-0.442	-0.289
	P Val.	0.040	0.236	0.009	0.159	0.009	0.092
	FDR	0.576	0.801	0.182	0.689	0.182	0.647
Lesion Load	Rho	-0.223	-0.168	-0.409	-0.243	-0.269	-0.234
	P Val.	0.147	0.259	0.006	0.101	0.112	0.163
	FDR	0.689	0.801	0.182	0.647	0.647	0.689

Table 8: Partial correlation analysis using Spearman's correlation coefficient comparing clinical metrics with both streamline bundle (*"Streamline Bun.*") load (volume of streamlines intersecting lesions divided by WM streamline bundle volume grouped by WM column) and lesion load (volume of lesions grouped by WM column divided by volume of WM column). FDR p-values are bolded if $p < 0.05$.

Spearman's Partial Correlation:
Streamline Bundle & Lesion Streamline FA vs. Clinical Metrics

		**EDSS**					
		Left Dorsal	Right Dorsal	Left Lateral	Right Lateral	Left Ventral	Right Ventral
Streamline Bundle FA	Rho	-0.020	-0.085	0.163	0.020	-0.144	-0.197
	P Val.	0.885	0.547	0.244	0.885	0.310	0.165
	FDR	0.936	0.787	0.607	0.936	0.685	0.528
Lesion Stream. FA	Rho	-0.076	-0.063	0.103	0.000	0.126	-0.058
	P Val.	0.612	0.671	0.501	0.997	0.476	0.739
	FDR	0.832	0.848	0.780	0.997	0.780	0.877
		**TUG**					
		Left Dorsal	Right Dorsal	Left Lateral	Right Lateral	Left Ventral	Right Ventral
Streamline Bundle FA	Rho	-0.062	-0.227	-0.122	-0.222	-0.207	-0.215
	P Val.	0.660	0.102	0.384	0.109	0.141	0.129
	FDR	0.848	0.528	0.734	0.528	0.528	0.528
Lesion Stream. FA	Rho	-0.056	-0.316	-0.161	-0.293	0.013	-0.421
	P Val.	0.709	0.029	0.291	0.044	0.941	0.012
	FDR	0.877	0.474	0.676	0.474	0.955	0.426
		**T25w**					
		Left Dorsal	Right Dorsal	Left Lateral	Right Lateral	Left Ventral	Right Ventral
Streamline Bundle FA	Rho	-0.094	-0.275	-0.141	-0.251	-0.199	-0.210
	P Val.	0.505	0.046	0.314	0.070	0.158	0.139
	FDR	0.78	0.474	0.685	0.528	0.528	0.528
Lesion Stream. FA	Rho	-0.096	-0.297	-0.149	-0.264	-0.063	-0.438
	P Val.	0.521	0.041	0.327	0.070	0.723	0.008
	FDR	0.782	0.474	0.693	0.528	0.877	0.426
		**Vibratron Threshold Left**					
		Left Dorsal	Right Dorsal	Left Lateral	Right Lateral	Left Ventral	Right Ventral
Streamline Bundle FA	Rho	-0.192	-0.093	-0.165	-0.022	-0.075	-0.096
	P Val.	0.168	0.509	0.237	0.874	0.599	0.505
	FDR	0.528	0.780	0.607	0.936	0.830	0.780
Lesion Stream. FA	Rho	-0.222	-0.235	-0.258	-0.128	-0.218	-0.018
	P Val.	0.133	0.107	0.087	0.387	0.216	0.917
	FDR	0.528	0.528	0.528	0.734	0.599	0.943
		**Vibratron Threshold Right**					
		Left Dorsal	Right Dorsal	Left Lateral	Right Lateral	Left Ventral	Right Ventral
Streamline Bundle FA	Rho	-0.066	-0.046	-0.185	-0.027	-0.198	-0.168
	P Val.	0.640	0.743	0.186	0.847	0.159	0.238
	FDR	0.848	0.877	0.558	0.936	0.528	0.607
Lesion Stream. FA	Rho	-0.093	-0.123	-0.250	-0.100	0.023	0.094
	P Val.	0.535	0.404	0.098	0.498	0.897	0.589

	FDR	0.786	0.746	0.528	0.781	0.936	0.830
		**Disease Duration**					
		Left Dorsal	Right Dorsal	Left Lateral	Right Lateral	Left Ventral	Right Ventral
Streamline Bundle FA	Rho	-0.033	-0.099	-0.064	-0.123	0.183	0.159
	P Val.	0.817	0.48	0.651	0.379	0.194	0.266
	FDR	0.933	0.78	0.848	0.734	0.559	0.637
Lesion Stream. FA	Rho	0.043	-0.136	0.106	0.027	0.242	0.379
	P Val.	0.773	0.357	0.489	0.857	0.169	0.025
	FDR	0.898	0.734	0.780	0.936	0.528	0.474

Table 9: Partial correlation analysis using Spearman's correlation coefficient comparing clinical metrics with both streamline bundle FA (average FA along streamlines grouped by WM column) and lesion streamline ("*Lesion Stream.*") FA (average FA along streamlines intersecting lesions grouped by WM column). FDR p-values are bolded if $p < 0.05$.
